# Yucasin Alleviates Aluminum Toxicity Associated with Regulating Reactive Oxygen Species Homeostasis in Tomato Seedlings

**DOI:** 10.3390/toxics13050406

**Published:** 2025-05-17

**Authors:** Huabin Liu, Chuangyang Bai, Jiahui Cai, Yue Wu, Changwei Zhu

**Affiliations:** 1 College of Biomedicine and Health, Anhui Science and Technology University, Chuzhou 233100, China; 18225784771@163.com (C.B.); caijh@ahstu.edu.cn (J.C.); 3241800830@njfu.edu.cn (Y.W.); 2 College of Life Sciences, Nanjing Forestry University, Nanjing 210037, China

**Keywords:** aluminum toxicity, yucasin, ROS homeostasis, antioxidant system, tomato

## Abstract

The phytotoxicity of aluminum (Al) to plants is well known. Auxin accumulation and reactive oxygen species (ROS) burst induced by Al toxicity are the key factors in root growth inhibition. Yucasin, an auxin synthesis inhibitor, effectively ameliorates Al phytotoxicity in tomato seedlings. However, the physiological mechanisms by which yucasin alleviates Al phytotoxicity in tomatoes remain elusive. Here, we examined the regulatory mechanisms of yucasin involved in tomato seedling growth under Al conditions through phenotypic, plant physiology analysis, and cellular experiments. Exogenous indole-3-acetic acid (IAA) application increased Al accumulation in tomato seedling roots, while yucasin decreased Al accumulation. Yucasin application reduced Al-induced ROS accumulation, lipid peroxidation, and cell death, enhanced root viability, and promoted tomato seedling root growth. Further, yucasin enhanced the antioxidant enzyme activities of superoxide dismutase, catalase, and peroxidase in plants under Al conditions. The results suggest that yucasin improves the scavenging capacity of ROS by maintaining the activities of antioxidative enzymes. This study elucidates the physiological mechanism by which yucasin alleviates Al phytotoxicity, highlighting its potential to enhance plant tolerance under acidic Al conditions.

## 1. Introduction

Al in the soil predominantly exists in the form of insoluble silicates or aluminum oxides, which cannot be absorbed by plants. Therefore, these forms of Al are less phytotoxic or non-phytotoxic to plants [1,2]. In acidic soils, Al forms facilitate conversion to soluble Al^3+^ and absorption by plants. Al^3+^ ions are phytotoxic to most plants [3]. The accumulation of excessive Al destroys the structure of the cell walls, affects cell division, inhibits root growth, and impacts water and nutrient absorption [4,5]. Therefore, Al has become a crucial limiting factor for plant growth and crop productivity in acidic arable land.

The plant root tips are the primary target site for Al^3+^, and the transition zone (TZ) is the key site where plants perceive Al toxicity signals [6,7]. Al stress triggers signal responses in the TZ, characterized by elevated ROS levels and perturbation of phytohormone profiles [8,9]. Auxin is a key signaling molecule for plants to respond to Al toxicity. Local auxin biosynthesis was enhanced, and auxin transport was disrupted under Al stress, resulting in auxin accumulation within the TZ [6,7,10,11]. This heightened auxin response amplifies the inhibitory effects of Al on root cell growth. Experimental evidence demonstrates that Al stress applied to the elongation zone (EZ) does not affect root growth; however, when Al is applied to the TZ, root growth is significantly inhibited [12]. The disparity in perception and response sites implies a distinct signaling mechanism or crosstalk between cells in the TZ and EZ. Recent evidence supports that auxin functions as a crucial intermediary in establishing the connection between these two zones. Exogenous boron restores auxin distribution in the EZ by re-establishing PIN2-mediated basipetal auxin transport from the TZ to EZ, thereby mitigating Al stress-induced inhibition of root growth [11]. Auxin levels in *Arabidopsis* are negatively correlated with their ability to tolerate Al. Mutants with excessive auxin synthesis (*yucca*, *sur2*, *sur1-3*) or exogenous application of auxin (NAA) aggravate sensitivity to Al stress [13]. Meanwhile, mutants with reduced auxin synthesis (*taa1*, *yuc8*, *yuc8/9*) or exogenous application of auxin synthesis inhibitors attenuate *Arabidopsis* sensitivity to Al stress [7,10]. Although multiple auxin biosynthesis pathways have been identified, the indole-3-pyruvate pathway has been established as the primary route for auxin biosynthesis in plants. In this pathway, the aminotransferase TAA and the flavin monooxygenase YUC sequentially catalyze the transformation of tryptophan into IAA [14]. Yucasin, a YUC inhibitor, is commonly used to investigate the regulatory mechanisms of auxin [10,15]. However, the physiological mechanisms by which auxin synthesis inhibitors alleviate Al toxicity in tomatoes remain unclear.

As an early stress response signal, ROS has been known to cope with Al toxicity by activating downstream gene expression or resistance systems in plants [16,17]. Al stress exposure stimulates ROS burst and accumulation in plant roots [18,19]. Excessive ROS oxidatively damage cells and protein structures, disrupt metabolic processes, and cause secondary damage to plants [19,20,21]. One of the main guarantees for protecting cells against oxidative damage is the activation of the antioxidant defense mechanism [22]. The antioxidant system balances plant growth and stress response by scavenging excessive ROS accumulation and maintaining ROS homeostasis. The transcription factor STOP1 is a key resistance factor to Al stress in plants, regulated by way of H_2_O_2_ to promote its oxidation and degradation and increase the sensitivity of plants to Al toxicity [23,24,25]. Studying the Al-sensitive mutant *rae6* showed that Al toxicity caused H_2_O_2_ accumulation in the mitochondria of the *rae6* mutant, and the phenotype of Al-sensitive *rae6* could be rescued by scavenging H_2_O_2_ [24]. Additionally, exogenous application of silicon and boron alleviates Al phytotoxicity by enhancing the antioxidant system and improving the ability to scavenge ROS [11,22,26]. These results implicate H_2_O_2_ accumulation as a crucial factor that contributes to plant susceptibility to Al stress.

Precious studies have confirmed that auxin and ROS are jointly involved in plant Al stress responses [27,28,29]. In the auxin transport-related mutant *osaux3*, auxin levels and ROS accumulation are significantly decreased [30]. Exogenous auxin application has been associated with enhanced ROS accumulation in root tips, and this effect is auxin concentration dependent [31]. In contrast, ROS alters the distribution and levels of auxin by affecting the polar localization of auxin transport carriers on the cell membrane, the oxidation and degradation of auxin, and ultimately controlling auxin-mediated plant growth [32,33,34]. These findings suggest an interaction between ROS and auxin to co-regulate the adaptive response process to plant Al stress. Nevertheless, the mechanisms underlying auxin-mediated plant growth by participating in ROS signaling regulation under Al stress are poorly understood [28,34].

Here, we revealed the regulatory mechanisms of yucasin responsible for tomato aluminum resistance through phenotypic, plant physiology analysis, and cellular experiments. The application of yucasin promotes root growth and significantly alleviates Al toxicity in tomatoes. Together, the findings provide insight into the potential of yucasin application in agricultural production to reduce the effect of Al phytotoxicity.

## 2. Materials and Methods

### 2.1. Plant Growth Conditions

The cherry tomato (*Solanum lycopersicum* L.) ‘Aisheng’ (Jingyan Yinong Seed Sci-Tech Co., Ltd., Beijing, China) was used in this study. After germination on 1/2 MS medium, tomato seeds were vertically cultured in a light incubator for 3 d. The seedlings were transferred to a 1/5 strength Hoagland hydroponic solution and adapted cultivation for one day. The tomato seedlings were held at 24 °C and 75% humidity under a 14/10 h light (10,000 Lux) cycle.

### 2.2. Treatment with AlCl_3_, IAA and Yucasin

At least 10 plant seedlings per group were pretreated with 0.5 mM CaCl_2_ for 12 h, transferred to a 1/5 strength Hoagland solution with 50 μM AlCl_3_, 50 μM AlCl_3_ plus 5 nM IAA or 5 μM yucasin for 6 d or the indicated times, respectively. The treatment solution (pH 5.0) was refreshed every two days.

### 2.3. Al Content Determination

The root system was obtained by cutting the treated tomato seedlings with a blade. After drying them, the samples were ground into a powder before being digested with HNO_3_-H_2_O_2_ [35]. Briefly, 5 mL of 65% HNO_3_ was added to the sample and incubated for 12 h, followed by boiling at 120 °C for 3 h. After cooling, 1 mL of H_2_O_2_ was added to make the solution transparent. The aluminum contents were measured using ICP-MS (NexION 2000, PerkinElmer, Waltham, MA, USA).

### 2.4. ROS Assays

The tomato seedlings were transferred to 1/5 Hoagland containing 50 μM AlCl_3_, or 50 μM AlCl_3_ + 5 μM yucasin for 1 day. After treatment, O_2_^−^ was detected using NBT staining in the roots. Briefly, the roots were rinsed with ddH_2_O and immersed in 0.25 mg/mL NBT. Roots were observed with a microscope. The NBT staining intensity was measured and analyzed using ImageJ (v1.51). Three biological replicates with 10–15 seedlings per treatment.

For H_2_O_2_ detection, 0.1 g of the treated fresh plant seedling was thoroughly ground into a homogenate with a mortar and pestle at 0 °C, and H_2_O_2_ was extracted by H_2_O_2_ content detection kit (BC3590, Solarbio, Beijing, China) following the instructions provided by the suppliers. The H_2_O_2_ content was determined at 415 nm.

### 2.5. Evaluation of Malondialdehyde (MDA) and Proline Content

To determine the MDA content, 0.5 g of the treated fresh tomato seedlings were homogenized in trichloroacetic acid (5%, *v*/*v*) with a mortar and pestle, and the supernatant was collected after centrifugation. The samples were incubated with 2 mL of 0.67% (*w*/*v*) TBA at 100 °C for 30 min, chilled, and collected for further analysis. TBA (0.67%) was dissolved in 10% trichloroacetic acid solution. The content of MDA was determined as described previously [36].

The proline content was measured as previously described [37]. The treated tomato seedling tissue was thoroughly ground in aqueous sulfosalicylic acid (3%, *w*/*v*), incubated at 100 °C for 10 min, and filtered after cooling. The sample filtrate, glacial acetic acid, and acid-ninhydrin (1:1:1, *v*/*v*/*v*) were reacted at 100 °C, chilled, and toluene was then introduced into the mixture for extraction before detecting the absorbance at 520 nm.

### 2.6. TTC and Evans Blue Staining

Root viability and cell death were detected by TTC [38] and Evans blue staining [39], respectively, as previously described. The treated tomato seedlings were immersed in 0.4% TTC for 4 h or 0.25% Evans blue in 10 mM PBS (pH 7.4) (P397924-5EA, Aladdin, Shanghai, China) for 5 min, respectively. Roots were observed with a light microscope. The TTC and Evans blue staining intensity was measured and analyzed using ImageJ (v1.51). Three biological replicates with 10–15 seedlings per treatment.

### 2.7. Enzyme Activity Assays

Approximately 0.5 g of the treated fresh plant seedling samples was collected and ground into a homogenate in phosphate buffer with a mortar and pestle. They were centrifuged, and the supernatant was used for the determination of enzyme activities. Superoxide dismutase and peroxidase activities in tomato seedlings were determined according to Yang et al. [40]. Catalase was determined using a CAT activity assay kit (BC0205, Solarbio, Beijing, China). CAT is the primary enzyme responsible for scavenging H_2_O_2_ and is extensively distributed across plant tissues and cells. H_2_O_2_ exhibits a characteristic absorption at 240 nm, and the absorbance decreases as it is decomposed by CAT. For the experiment, 0.1 g of the treated fresh tomato seedlings was thoroughly homogenized in a mortar and pestle. The resulting homogenate was transferred to a centrifuge tube, and 1 mL of phosphate buffer was added. After centrifugation at 8000× *g* for 10 min, the supernatant containing the CAT was collected. The reaction solution consisted of the supernatant and H_2_O_2_, and the absorbance was detected at 240 nm using a UV-2600 spectrophotometer (SHIMADZU, Suzhou, China). The enzymatic activity of CAT was subsequently evaluated based on the change in absorbance of the reaction solution.

### 2.8. Statistical Analysis

Data were analyzed by one-way ANOVA using PASW statistics 18. Duncan’s multi-comparison test was employed to assess statistical differences between various treatments at the *p* < 0.05 level.

## 3. Results

### 3.1. Yucasin Enhances Tomato Seedling Tolerance to Al Stress by Maintaining Root Viability

**Figure 1:**
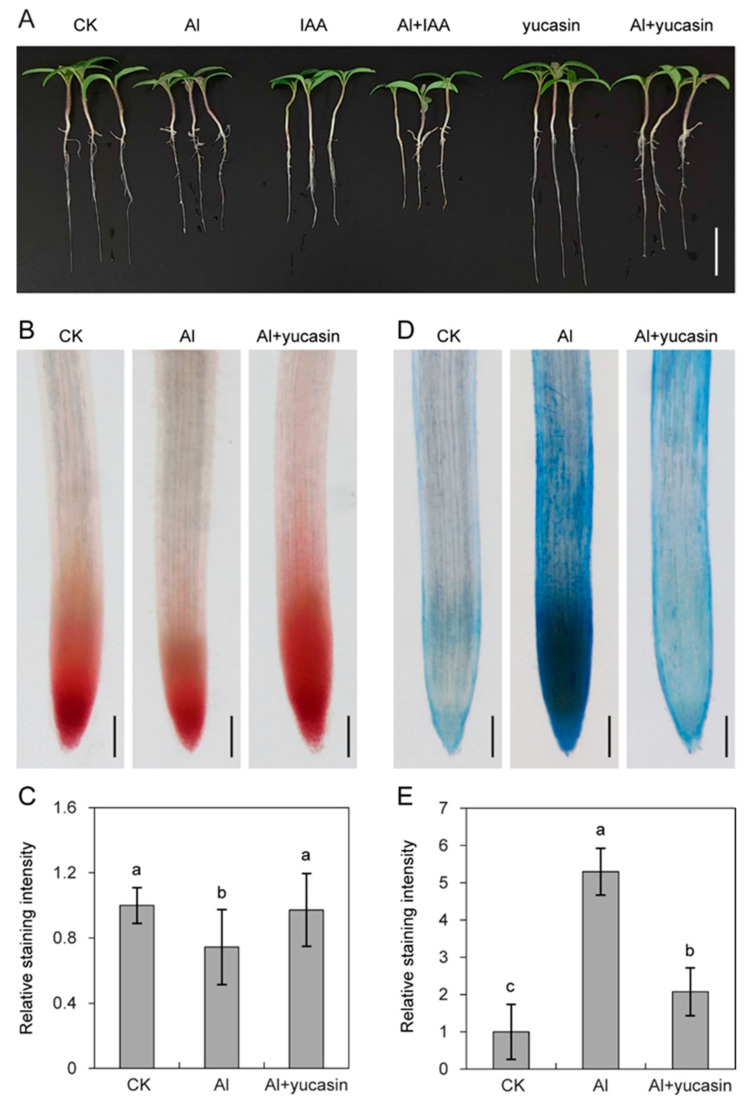
Yucasin promotes root growth under Al conditions by increasing root viability and reducing cell death. (**A**) Phenotypes of tomato seedlings after 6 d under 50 μM AlCl_3_ (Al), 5 nM IAA (IAA), 50 μM AlCl_3_ + 5 nM IAA (Al + IAA), 5 μM yucasin (yucasin), 50 μM AlCl_3_ + 5 μM yucasin (Al + yucasin) treatment as indicated. (**B**,**D**) TTC (**B**) staining for root activity and Evans blue (**D**) staining for cell death in the tomato seedlings exposed or not to 50 μM AlCl_3_ or 50 μM AlCl_3_ + 5 μM yucasin for 48 h. (**C**,**E**) indicated quantification analyses of staining intensities in the roots of tomato seedlings in (**B**,**D**), respectively. Error bars indicate SD (*n* = 10–15 per treatment on each replicate). Bars = 1 cm (**A**) and 200 μm (**B**,**D**). Different letters indicate significant differences.

Auxin has been recognized as a regulator of plant responses to Al stress [41]. To investigate the functions of auxin in plant Al toxicity, the seedlings were co-treated with IAA or auxin synthesis inhibitor yucasin in the presence of AlCl_3_. IAA enhanced Al-induced root growth inhibition, while yucasin promoted tomato root elongation (Figure 1A, Figures S1 and S2). This indicates that yucasin plays positive regulatory roles in tomato seedling root growth under Al toxicity.

Maintaining the cell activity of root tips is the main driver promoting plant root elongation. To analyze the roles of yucasin on the root activity in tomatoes under Al toxicity, cell viability and cell death were assessed by histochemical staining. As shown in Figure 1, the TTC staining intensity was significantly reduced, while the Evans blue staining intensity was significantly enhanced in tomato seedling roots exposed to Al stress (Figure 1B–E). This indicates that Al stress induced cell death and reduced cell viability. Consistent with the root growth phenotype, the effects of Al toxicity were reversed by applying yucasin. Compared to tomato seedlings exposed to Al stress, adding yucasin increased root activity and reduced cell death (Figure 1B–E). These results suggested that yucasin contributes to the Al detoxification and tolerance of tomato seedlings by enhancing root activity and reducing cell death.

### 3.2. Yucasin Reduces Al Accumulation in Tomato Seedling Roots

**Figure 2:**
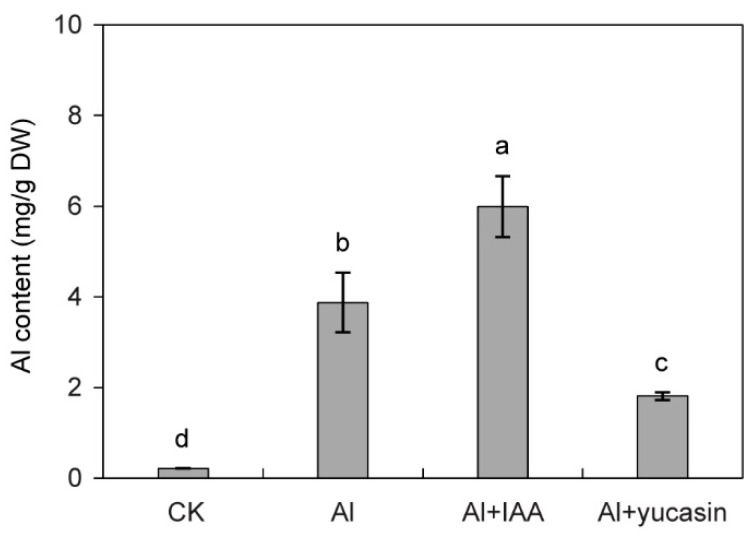
Effects of IAA and yucasin on Al concentration in tomato seedling roots under Al stress. Quantification analysis of Al^3+^ content in the tomato seedlings exposed or not to 50 μM AlCl_3_ (Al), 50 μM AlCl_3_ + 10 nM IAA (Al + IAA), 50 μM AlCl_3_ + 1 μM yucasin (Al + yucasin) treatment as indicated for 6 d (mean ± SD, *n* = 3). Letters above the columns indicate statistically different (ANOVA followed by Duncan test).

Al^3+^ is absorbed by the roots and accumulates in the cell wall and cytoplasm, thereby disrupting cellular physiological processes and inhibiting cell division, which ultimately suppresses plant growth. To analyze whether auxins affect the uptake of Al^3+^ in tomato, we used ICP-MS to analyze the aluminum ion levels in tomato seedling roots treated with exogenous auxin IAA and yucasin. Treatment with IAA led to an enhanced accumulation of Al^3+^ in tomato seedling roots (Figure 2). Conversely, applying yucasin decreases such aluminum ion accumulation (Figure 2). These findings suggest that IAA facilitates the absorption of Al^3+^ by tomato roots, whereas yucasin impedes the root absorption of aluminum ions.

### 3.3. Yucasin Reduces ROS Accumulation and Oxidative Damage Under Al Toxicity

**Figure 3:**
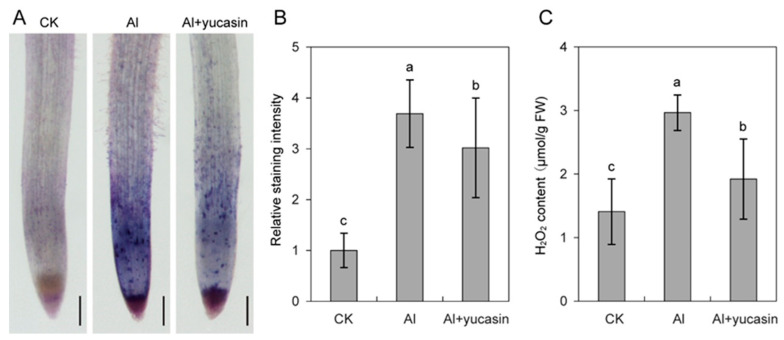
Yucasin reduces Al-induced ROS accumulation in seedlings. (**A**) NBT staining for O_2_^–^ in roots of tomato seedling exposed or not to AlCl_3_, AlCl_3_ (Al) plus yucasin (Al + yucasin) for 24 h. (**B**) Quantification analysis of staining intensities (**A**) in the roots of tomato seedlings in (**B**). Bars = 200 μm. Values represent the means ± SD ((**A**,**B**): *n* = 10–15 per treatment on each replicate). (**C**) Quantification of the yucasin effect on H_2_O_2_ level in the tomatoes under control (CK), Al, and Al + yucasin conditions. Error bars indicate SD (*n* = 3). Letters above the columns indicate statistically different (ANOVA followed by Duncan test).

ROS include H_2_O_2_ and O_2_^−^, important signals for plants to respond to abiotic stresses. The appropriate level of ROS signal mediates plant response to stress by activating downstream resistance genes. Meanwhile, excessive ROS accumulation causes oxidative stress to plants, exacerbating the damage of stress to plants. To investigate whether yucasin mitigates Al toxicity in tomato seedlings by modulating ROS production, we analyzed the H_2_O_2_ content in tomato seedlings and monitored the level and distribution of O_2_^−^ in tomato root tips using NBT staining. As shown in Figure 3, Al treatment triggers ROS production in tomato seedlings. ROS bursts mainly occurred in the meristematic and transitional zones of the tomato roots. In contrast, exogenous yucasin application significantly reduced ROS accumulation (Figure 3). The results suggest that plant root tips are the primary target sites for Al^3+^, while yucasin mitigates Al phytotoxicity by regulating Al-induced ROS accumulation in seedlings.

**Figure 4:**
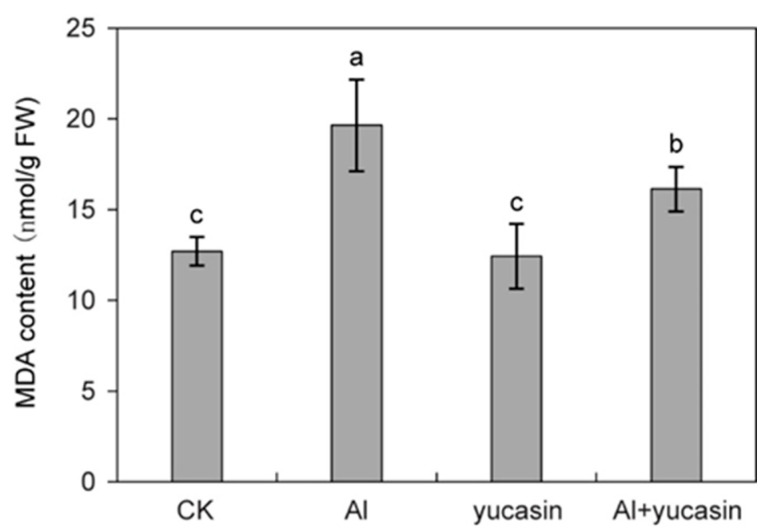
Yucasin alleviates Al-induced oxidative stress in tomato seedlings. Quantification of MDA content in the tomato seedlings exposed or not to AlCl_3_, yucasin, and AlCl_3_ + yucasin for 6 d (mean ± SD, *n* = 3). Letters above the columns indicate statistically different (ANOVA followed by Duncan test).

**Figure 5:**
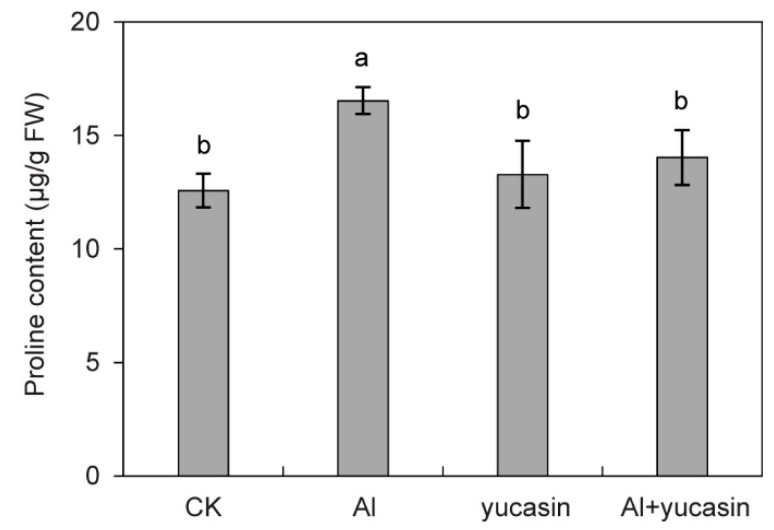
Effect of yucasin on proline level in Al-treated tomato seedlings. Quantification analysis of proline content in the tomato seedlings exposed or not to AlCl_3_, yucasin, and AlCl_3_ + yucasin for 6 d (mean ± SD, *n* = 3). Letters above the columns indicate statistically different (ANOVA followed by Duncan’s test).

MDA is a byproduct of lipid peroxidation in cell membranes under oxidative stress conditions. Its content is an indicator of the extent of plasma membrane oxidative damage. To evaluate oxidative damage induced by Al stress, the MDA levels in tomato seedlings were analyzed. Consistent with the ROS accumulation, the MDA content in tomatoes significantly increased under Al stress (Figure 4). When yucasin was applied, the accumulation of MDA content caused by Al stress was significantly reduced (Figure 4). These results suggested that yucasin alleviates Al toxicity by reducing ROS accumulation and peroxidation damage. Proline is an important regulatory substance in plants’ response to stress. It has the functions of stabilizing the membrane structure and regulating cell osmotic potential. Tomato seedlings exposed to Al stress showed an increase in proline content (Figure 5). Yucasin application reduced Al-induced proline accumulation (Figure 5).

### 3.4. Yucasin Maintains Antioxidant Enzyme Activities in Tomato Seedlings

**Figure 6:**
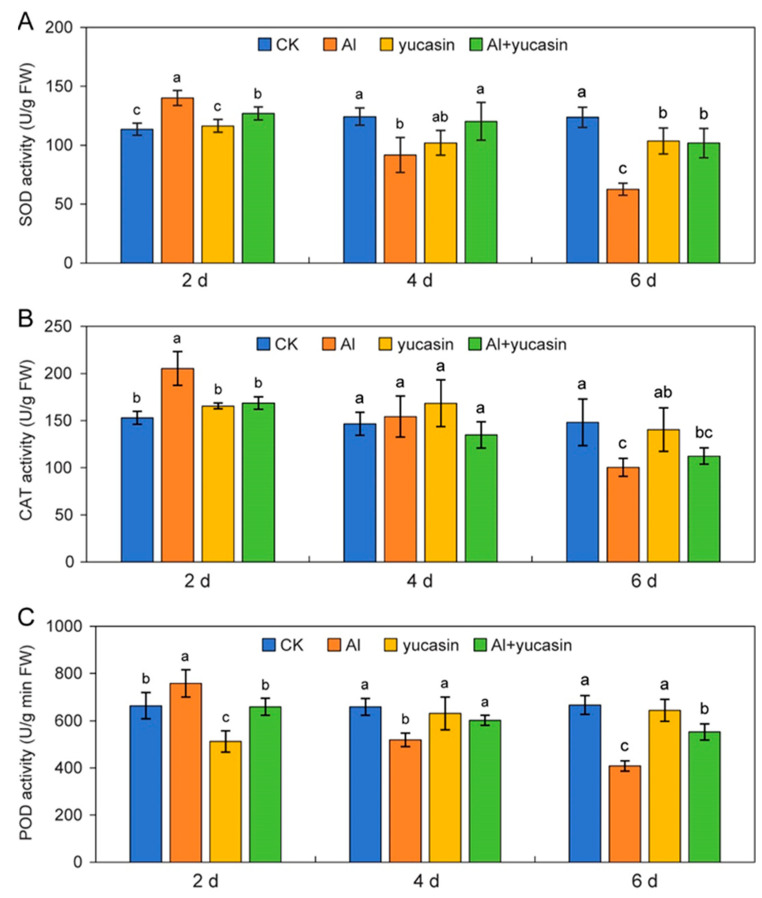
Effects of yucasin on antioxidant enzyme activities. (**A**–**C**) Quantification analysis of the activities of SOD (**A**), CAT (**B**), and POD (**C**) in the tomato seedlings exposed or not (CK) to AlCl_3_ (Al), yucasin (yucasin), and AlCl_3_ + yucasin (Al + yucasin) for 2, 4, and 6 d (mean ± SD, *n* = 3). Different letters within an indicated time point represent significant differences among the control (CK), Al, yucasin, and Al + yucasin treatments (ANOVA followed by Duncan’s test).

Maintaining the dynamic balance of ROS by scavenging excessively accumulated ROS is an important regulatory mechanism to protect plants from oxidation stress. It was shown that AlCl_3_ treatments resulted in an elevation of ROS levels in tomato seedlings, with a notable O_2_^–^ accumulation observed in root tips (Figure 3A–C). Plants cope with oxidative stress by regulating the activities of ROS-scavenging enzymes, which include SOD, POD, and CAT. As depicted in Figure 6, the activities of SOD, POD, and CAT in tomato seedlings exhibited a trend of initially increasing and subsequently decreasing during Al toxicity (Figure 6A–C). Two days after Al treatment, the activities of these enzymes significantly increased (Figure 6A–C). With the prolongation of AlCl_3_ treatment (4 or 6 d), the enzyme activities significantly decreased (Figure 6A–C). This indicated that plants cope with excessive ROS production by increasing the antioxidant enzyme activity within the early stage of Al conditions. However, with the prolongation of Al treatment, the antioxidant enzyme activity in plants is significantly inhibited (Figure 6A–C). After 6 d of Al treatment, the SOD, CAT, and POD activity significantly decreased (Figure 6A–C). Applying yucasin for 6 d to the Al-treated tomato seedlings increased the SOD, CAT, and POD activity by 62.5%, 12%, and 35.4%, respectively, compared to the Al treatments (Figure 6A–C).

## 4. Discussion

Plants coping with environmental stress is a complex growth decision that is influenced and regulated by many factors, among which auxin plays a central role. The mechanisms by which auxin inhibits or promotes plant growth under acid-aluminium stress vary considerably among plant species. Auxin biosynthesis mutants *yuc9*, *taa1-1*, and *yucQ* of *Arabidopsis* exhibit Al tolerance [7,10], while auxin synthesis excess mutants (*yucca*, *sur2*, *sur1-3*) show sensitivity to Al stress [13]. Consistent with the results, we found that treatment with exogenous auxin IAA increased the sensitivity of tomatoes to Al stress, whereas yucasin significantly reduced the sensitivity (Figure 1 and Figure S1). These results indicate that plant Al tolerance is inversely proportional to auxin levels. However, there are contrary experimental findings that Al stress inhibits maize root growth, accompanied by a significant decrease in auxin content [42]. Exogenous application of auxin analog NAA can alleviate Al-induced maize root growth inhibition [42]. Al, which is phytotoxic to most plants, becomes a beneficial element in *Camellia sinensis* growth [43]. Al stimulates plant growth and is an essential element for maintaining root development in *Camellia sinensis* [44]. Al-induced UDP glycosyltransferase CsUGT84J2 promotes root growth in *Camellia sinensis* by enhancing the accumulation of auxin [45]. These findings suggest the existence of distinct regulatory mechanisms in various plant species under Al conditions.

Certain plants exhibit resilience in aluminum-rich environments, with some even experiencing growth enhancement under low Al concentrations [44,45]. This phenomenon is attributed to the evolutionary development of regulatory mechanisms in Al-tolerant plants to combat Al stress. The mechanisms include the secretion of organic acids to chelate Al^3+^, elevation of pectin methylation in cell walls, and sequestration of Al^3+^ within vacuoles to mitigate Al toxicity [46,47,48]. Pectin, a key constituent of plant cell walls, possesses a high affinity for Al^3+^ [49]. The accumulation of Al damages cell wall integrity, disrupts cell division, and suppresses cellular growth. Here, we showed that applying IAA promoted the uptake of Al^3+^ by the tomato seedling roots. Meanwhile, yucasin inhibited the acquisition of aluminum ions by roots (Figure 2). Therefore, reducing the absorption of Al^3+^ by roots and alleviating the pressure induced by Al stress may be crucial factors for yucasin in mitigating Al toxicity.

ROS production and signaling serve as crucial regulatory mechanisms that enable plants to cope with environmental challenges and establish defense responses [33]. However, excessive ROS production can impose oxidative stress on plant cells, trigger oxidative damage, and lead to cell death. We found that aluminum stress triggered ROS overproduction in tomato seedlings (Figure 3). The accumulated ROS inflicted oxidative damage on plant cells. This was manifested by an elevation in MDA levels and a decline in cell viability (Figure 1B and Figure 4). When yucasin was applied, the Al-induced ROS accumulation and MDA content decreased (Figure 3 and Figure 4). Consistently, 4-phenoxyphenylboronic acid (PPBo), another inhibitor of auxin biosynthesis, reduces H_2_O_2_ accumulation caused by mild cadmium stress and alleviates the phytotoxicity on rice and barley [50,51]. These results suggest that yucasin and PPBo exhibit comparable functions in reducing ROS accumulation and ameliorating metal stress in plants. However, PPBo does not affect the absorption of cadmium ions by the roots, while yucasin reduces Al accumulation in the tomato seedlings, which may be attributed to distinct mechanisms of action or differences in treatment conditions and experimental materials.

Although it has been established that both auxin and ROS are involved in the Al stress response, their roles in mitigating Al toxicity in plants, especially the regulatory interplay between auxin and ROS, remain poorly understood [28]. Exogenous auxin triggers the production and accumulation of ROS, leading to tomato root growth inhibition [31]. Applying yucasin significantly reduced Al-induced ROS accumulation and promoted root elongation (Figure 3). The results indicate that auxin regulates root growth by affecting ROS levels. However, there is a ROS-auxin-mediated signaling pathway in rice that promotes root elongation in the stress-free area to avoid adverse stress [33]. These results suggest that there is a complex interaction between auxin and ROS involved in plant stress response.

Adverse stresses induce a burst of ROS and disrupt the ROS scavenging system, consequently exacerbating plant stress damage [52]. The ROS level within plant cells is strictly regulated by the antioxidant detoxification systems to protect the plant from the oxidative stresses caused by excessive ROS accumulation. Studies have demonstrated that the application of chemicals, such as silicon and boron, significantly improves plant tolerance to Al stress by enhancing ROS-scavenging ability [11,22]. This, in turn, substantially expands our understanding of the use of exogenous substances for mitigating Al phytotoxicity. Consistently, it was found that yucasin application markedly reduced the contents of H_2_O_2_, O_2_^−^, and MDA and enhanced antioxidant enzymes activities under AlCl_3_ treatments (Figure 6). The maintenance or enhancement of the antioxidant enzyme system is regarded as a key strategy for ROS scavenging and an essential regulatory mechanism for mitigating plant stress [53,54]. The results suggest that yucasin enhances ROS-scavenging ability by increasing antioxidant enzyme activities and mitigating oxidative stresses induced by Al toxicity. This provides novel evidence for further elucidation of the aluminum detoxification mechanism of yucasin. However, further investigation is needed to determine whether the reduction in ROS under Al conditions is attributable to the exogenous application of yucasin or a decrease in auxin levels.

## 5. Conclusions

In this study, tomato seedlings were employed to investigate the regulatory mechanism underlying plants’ response and tolerance to Al conditions. A burst of ROS is a prominent feature of tomato seedlings responding to Al toxicity. With the prolongation of Al treatment, root vitality decreases, and root growth is inhibited. The application experiment of yucasin on tomato seedlings demonstrated that yucasin positively regulates plant aluminum tolerance by maintaining antioxidant enzyme activities to enhance ROS scavenging ability. Moreover, yucasin also reduced Al accumulation in tomato seedlings, further alleviating Al-induced stresses. The present study elucidates the physiological mechanisms by which yucasin mitigates Al toxicity in tomatoes, providing a potential strategy for utilizing yucasin in acid soils to improve plant tolerance to Al stress.

## Data Availability

Data is available in the article.

## Supplementary Materials

The following supporting information can be downloaded at: https://www.mdpi.com/article/10.3390/toxics13050406/s1, Figure S1: Effects of IAA, yucasin and Al stress on the seedling growth of tomatoes. Figure S2: Yucasin promotes root elongation under Al stress.

## References

[1] Kinraide T.B. (1991). Identity of the rhizotoxic aluminium species. Plant Soil.

[2] Ofoe R., Thomas R.H., Asiedu S.K., Wang-Pruski G., Fofana B., Abbey L. (2023). Aluminum in plant: Benefits, toxicity and tolerance mechanisms. Front. Plant Sci..

[3] Silva T.F., Ferreira B.G., Dos Santos Isaias R.M., Alexandre S.S., França M.G.C. (2020). Immunocytochemistry and density functional theory evidence the competition of aluminum and calcium for pectin binding in urochloa decumbens roots. Plant Physiol. Biochem..

[4] Jaskowiak J., Tkaczyk O., Slota M., Kwasniewska J., Szarejko I. (2018). Analysis of aluminum toxicity in Hordeum vulgare roots with an emphasis on DNA integrity and cell cycle. PLoS ONE.

[5] Zhang H., Jiang Z., Qin R., Zhang H., Zou J., Jiang W., Liu D. (2014). Accumulation and cellular toxicity of aluminum in seedling of *Pinus massoniana*. BMC Plant Biol..

[6] Li C., Liu G., Geng X., He C., Quan T., Hayashi K.I., De Smet I., Robert H.S., Ding Z., Yang Z.B. (2021). Local regulation of auxin transport in root-apex transition zone mediates aluminium-induced Arabidopsis root-growth inhibition. Plant J..

[7] Yang Z.B., Geng X., He C., Zhang F., Wang R., Horst W.J., Ding Z. (2014). TAA1-regulated local auxin biosynthesis in the root-apex transition zone mediates the aluminum-induced inhibition of root growth in Arabidopsis. Plant Cell.

[8] Ranjan A., Sinha R., Sharma T.R., Pattanayak A., Singh A.K. (2021). Alleviating aluminum toxicity in plants: Implications of reactive oxygen species signaling and crosstalk with other signaling pathways. Physiol. Plant.

[9] Yang Z.B., Liu G., Liu J., Zhang B., Meng W., Müller B., Hayashi K.I., Zhang X., Zhao Z., De Smet I. (2017). Synergistic action of auxin and cytokinin mediates aluminum-induced root growth inhibition in Arabidopsis. EMBO Rep..

[10] Liu G., Gao S., Tian H., Wu W., Robert H.S., Ding Z. (2016). Local transcriptional control of YUCCA regulates auxin promoted root-growth inhibition in response to aluminium stress in Arabidopsis. PLoS Genet..

[11] Tao L., Xiao X., Huang Q., Zhu H., Feng Y., Li Y., Li X., Guo Z., Liu J., Wu F. (2023). Boron supply restores aluminum-blocked auxin transport by the modulation of PIN2 trafficking in the root apical transition zone. Plant J..

[12] Kollmeier M., Felle H.H., Horst W.J. (2000). Genotypical differences in aluminum resistance of maize are expressed in the distal part of the transition zone. Is reduced basipetal auxin flow involved in inhibition of root elongation by aluminum?. Plant Physiol..

[13] Zhu X.F., Lei G.J., Wang Z.W., Shi Y.Z., Braam J., Li G.X., Zheng S.J. (2013). Coordination between apoplastic and symplastic detoxification confers plant aluminum resistance. Plant Physiol..

[14] Mashiguchi K., Tanaka K., Sakai T., Sugawara S., Kawaide H., Natsume M., Hanada A., Yaeno T., Shirasu K., Yao H. (2011). The main auxin biosynthesis pathway in Arabidopsis. Proc. Nat. Acad. Sci. USA.

[15] Nishimura T., Hayashi K., Suzuki H., Gyohda A., Takaoka C., Sakaguchi Y., Matsumoto S., Kasahara H., Sakai T., Kato J. (2014). Yucasin is a potent inhibitor of YUCCA, a key enzyme in auxin biosynthesis. Plant J..

[16] Fu X.Z., Wang X., Liu J.J., Chen Y.X., Wang A.Q., Zhan J., Han Z.Q., He L.F., Xiao D. (2025). AhASRK1, a peanut dual-specificity kinase that activates the Ca^2+^-ROS-MAPK signalling cascade to mediate programmed cell death induced by aluminium toxicity via ABA. Plant Physiol. Biochem..

[17] Ding Z.J., Xu C., Yan J.Y., Wang Y.X., Cui M.Q., Yuan J.J., Wang Y.N., Li G.X., Wu J.X., Wu Y.R. (2024). The LRR receptor-like kinase ALR1 is a plant aluminum ion sensor. Cell Res..

[18] Matsumoto H., Motoda H. (2013). Oxidative stress is associated with aluminum toxicity recovery in apex of pea root. Plant Soil.

[19] Huang W.J., Yang X.D., Yao S.C., LwinOo T., He H.Y., Wang A.Q., Li C.Z., He L.F. (2014). Reactive oxygen species burst induced by aluminum stress triggers mitochondria-dependent programmed cell death in peanut root tip cells. Plant Physiol. Bioch..

[20] Yamamoto Y., Kobayashi Y., Devi S.R., Rikiishi S., Matsumoto H. (2002). Aluminum toxicity is associated with mitochondrial dysfunction and the production of reactive oxygen species in plant cells. Plant Physiol..

[21] Jiang D., Ou Y., Jiang G., Dai G., Liu S., Chen G. (2025). Melatonin-priming ameliorates aluminum accumulation and toxicity in rice through enhancing aluminum exclusion and maintaining redox homeostasis. Plant Physiol. Biochem..

[22] Wang S., Cheng H., Wei Y. (2024). Supplemental silicon and boron alleviates aluminum-induced oxidative damage in soybean roots. Plants.

[23] Zhang Y., Zhang J., Guo J., Zhou F., Singh S., Xu X., Xie Q., Yang Z., Huang C.F. (2019). F-box protein RAE1 regulates the stability of the aluminum-resistance transcription factor STOP1 in Arabidopsis. Proc. Natl. Acad. Sci. USA.

[24] Wei X., Zhu Y., Xie W., Ren W., Zhang Y., Zhang H., Dai S., Huang C.F. (2024). H_2_O_2_ negatively regulates aluminum resistance via oxidation and degradation of the transcription factor STOP1. Plant Cell.

[25] Huang C.F., Ma Y. (2025). Aluminum resistance in plants: A critical review focusing on STOP1. Plant Commun..

[26] Huang L., Li H., Luo Y., Shi J., Kong L., Teng W. (2024). Exogenous silicon alleviates aluminum stress in Eucalyptus species by enhancing the antioxidant capacity and improving plant growth and tolerance quality. BMC Plant Biol..

[27] Wu D., Shen H., Yokawa K., Baluška F. (2014). Alleviation of aluminium-induced cell rigidity by overexpression of OsPIN2 in rice roots. J. Exp. Bot..

[28] Yuan H.M., Liu W.C., Jin Y., Lu Y.T. (2013). Role of ROS and auxin in plant response to metal-mediated stress. Plant Signal Behav..

[29] Bai B., Bian H., Zeng Z., Hou N., Shi B., Wang J., Zhu M., Han N. (2017). miR393-mediated auxin signaling regulation is involved in root elongation inhibition in response to toxic aluminum stress in Barley. Plant Cell Physiol..

[30] Wang M., Qiao J., Yu C., Chen H., Sun C., Huang L., Li C., Geisler M., Qian Q., Jiang A. (2019). The auxin influx carrier, OsAUX3, regulates rice root development and responses to aluminium stress. Plant Cell Environ..

[31] Ivanchenko M.G., den Os D., Monshausen G.B., Dubrovsky J.G., Bednárová A., Krishnan N. (2013). Auxin increases the hydrogen peroxide (H_2_O_2_) concentration in tomato (*Solanum lycopersicum*) root tips while inhibiting root growth. Ann. Bot..

[32] Shen T., Jia N., Wei S., Xu W., Lv T., Bai J., Li B. (2022). Mitochondrial HSC70-1 regulates polar auxin transport through ROS homeostasis in Arabidopsis roots. Antioxidants.

[33] Wang H.Q., Zhao X.Y., Xuan W., Wang P., Zhao F.J. (2023). Rice roots avoid asymmetric heavy metal and salinity stress via an RBOH-ROS-auxin signaling cascade. Mol. Plant.

[34] Ranjan A., Sinha R., Lal S.K., Bishi S.K., Singh A.K. (2021). Phytohormone signalling and cross-talk to alleviate aluminium toxicity in plants. Plant Cell Rep..

[35] Li L., Fu Q.L., Achal V., Liu Y. (2015). A comparison of the potential health risk of aluminum and heavy metals in tea leaves and tea infusion of commercially available green tea in Jiangxi, China. Environ. Monit. Assess..

[36] Lv W.T., Lin B., Zhang M., Hua X.J. (2011). Proline accumulation is inhibitory to Arabidopsis seedlings during heat stress. Plant Physiol..

[37] Bates L.S., Waldren R.P., Teare I.D. (1973). Teare. Rapid determination of free proline for water-stress studies. Plant Soil.

[38] Islam E., Yang X., Li T., Liu D., Jin X., Meng F. (2007). Effect of Pb toxicity on root morphology, physiology and ultrastructure in the two ecotypes of *Elsholtzia argyi*. J. Hazard. Mater..

[39] Motoda H., Kano Y., Hiragami F., Kawamura K., Matsumoto H. (2010). Morphological changes in the apex of pea roots during and after recovery from aluminium treatment. Plant Soil.

[40] Yang W., Wen D., Yang Y., Li H., Yang C., Yu J., Xiang H. (2024). Metabolomics and transcriptomics combined with physiology reveal key metabolic pathway responses in tobacco roots exposed to NaHS. BMC Plant Biol..

[41] Sun P., Tian Q.Y., Chen J., Zhang W.H. (2010). Aluminium-induced inhibition of root elongation in Arabidopsis is mediated by ethylene and auxin. J. Exp. Bot..

[42] Zhang M., Lu X., Li C., Zhang B., Zhang C., Zhang X.S., Ding Z. (2018). Auxin efflux carrier ZmPGP1 mediates root growth inhibition under aluminum stress. Plant Physiol..

[43] Pilon-Smits E.A., Quinn C.F., Tapken W., Malagoli M., Schiavon M. (2009). Physiological functions of beneficial elements. Curr. Opin. Plant Biol..

[44] Sun L., Zhang M., Liu X., Mao Q., Shi C., Kochian L.V., Liao H. (2020). Aluminium is essential for root growth and development of tea plants (*Camellia sinensis*). J. Integr. Plant Biol..

[45] Jiang X., Lai S., Kong D., Hou X., Shi Y., Fu Z., Liu Y., Gao L., Xia T. (2023). Al-induced CsUGT84J2 enhances flavonol and auxin accumulation to promote root growth in tea plants. Hortic. Res..

[46] Liu J.P., Magalhaes J.V., Shaff J., Kochian L.V. (2009). Aluminum-activated citrate and malate transporters from the MATE and ALMT families function independently to confer Arabidopsis aluminum tolerance. Plant J..

[47] Kochian L.V., Pineros M.A., Liu J.P., Magalhaes J.V. (2015). Plant adaptation to acid soils: The molecular basis for crop aluminum resistance. Annu. Rev. Plant Biol..

[48] Zhang F., Yan X., Han X., Tang R., Chu M., Yang Y., Yang Y.H., Zhao F., Fu A., Luan S. (2019). A defective vacuolar proton pump enhances aluminum tolerance by reducing vacuole sequestration of organic acids. Plant Physiol..

[49] Li X., Li Y., Qu M., Xiao H., Feng Y., Liu J., Wu L., Yu M. (2016). Cell wall pectin and its methyl-esterification in transition zone determine Al resistance in cultivars of pea (*Pisum sativum*). Front. Plant Sci..

[50] Demecsová L., Zelinová V., Liptáková L., Tamás L. (2020). Mild cadmium stress induces auxin synthesis and accumulation, while severe cadmium stress causes its rapid depletion in barley root tip. Environ. Exp. Bot..

[51] Guo L., Yang S., Tu Z., Yu F., Qiu C., Huang G., Fang S. (2024). An indole-3-acetic acid inhibitor mitigated mild cadmium stress by suppressing peroxide formation in rice seedling roots. Plant Physiol. Biochem..

[52] Tamás L., Mistrík I., Zelinová V. (2017). Heavy metal-induced reactive oxygen species and cell death in barley root tip. Environ. Exp. Bot..

[53] Li B., Fu Y., Li X., Yin H., Xi Z. (2022). Brassinosteroids alleviate cadmium phytotoxicity by minimizing oxidative stress in grape seedlings: Toward regulating the ascorbate-glutathione cycle. Sci. Hortic..

[54] Niu K., Zhu R., Wang Y., Zhao C., Ma H. (2023). 2,4-epibrassinolide improves cadmium tolerance and lateral root growth associated with regulating endogenous auxin and ethylene in *Kentucky bluegrass*. Ecotoxicol. Environ. Saf..

