# Supplementary material for: Yucasin Alleviates Aluminum Toxicity Associated with Regulating Reactive Oxygen Species Homeostasis in Tomato Seedlings
Source: Toxics. 2025 May 17;13(5):406. doi: 10.3390/toxics13050406 (PMC12115678; doi:10.3390/toxics13050406)
Supplement: Supplementary file 1 [file toxics-13-00406-s001.zip › toxics-3583982-supplementary.pdf]

Supplementary Figures

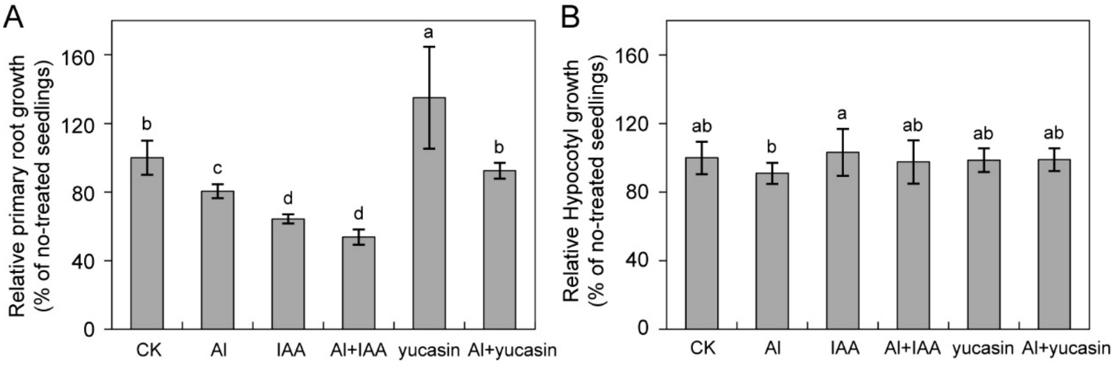

Figure S1. Effects of IAA, yucasin, and Al stress on the seedling growth of tomatoes

(A,B) The relative primary root (A) and hypocotyl (B) length of tomato seedlings after 6 d exposure to 50 μM $AlCl_3$, 5 nM IAA, 50 μM $AlCl_3$ plus 5 nM IAA, 5μM yucasin, or 50 μM $AlCl_3$ plus 5 μM yucasin.

Data are means ± SD (n=11). Data were analyzed by ANOVA followed by Duncan's test. Letters above the columns indicate significantly different.

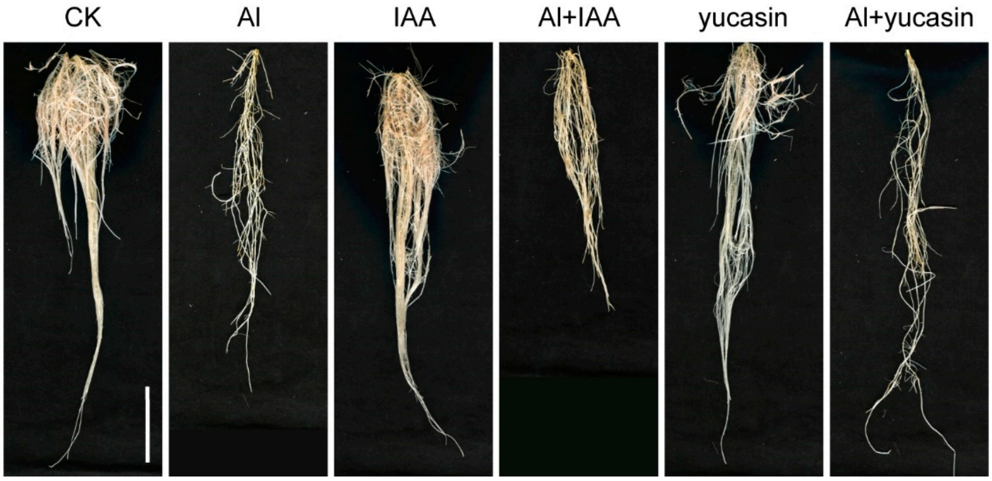

Figure S2. Yucasin promotes root elongation under Al stress

Phenotypes of tomato seedlings after 4 weeks under 50 μM $AlCl_3$, 10 nM IAA, 50 μM $AlCl_3$+10 nM IAA, 1 μM yucasin, 50 μM $AlCl_3$+1 μM yucasin treatment as indicated. Bars=5 cm.
